# Evaluation of a High Throughput Method for the Detection of Mutations Associated with Thrombosis and Hereditary Hemochromatosis in Brazilian Blood Donors

**DOI:** 10.1371/journal.pone.0125460

**Published:** 2015-05-08

**Authors:** Vivian Dionisio Tavares Niewiadonski, Juliana Vieira dos Santos Bianchi, Cesar de Almeida-Neto, Nelson Gaburo, Ester Cerdeira Sabino

**Affiliations:** 1 Institute of Tropical Medicine, University of São Paulo, São Paulo, Brazil; 2 Department of Molecular Diagnostics, DASA, São Paulo, Brazil; 3 Department of Infectious Disease, University of Sao Paulo, São Paulo, Brazil; 4 Fundação Pró-Sangue Hemocentro de São Paulo, São Paulo, Brazil; Icahn School of Medicine at Mount Sinai, UNITED STATES

## Abstract

**Background:**

The aim of this study was to evaluate the OpenArray platform for genetic testing of blood donors and to assess the genotype frequencies of nucleotide-polymorphisms (SNPs) associated with venous thrombosis (G1691A and G20210A), hyperhomocysteinemia (C677T, A1298C), and hereditary hemochromatosis (C282Y, H63D and S65C) in blood donors from Sao Paulo, Brazil.

**Methods:**

We examined 400 blood donor samples collected from October to November 2011. The SNPs were detected using OpenArray technology. The blood samples were also examined using a real-time PCR–FRET system to compare the results and determine the accuracy of the OpenArray method.

**Results:**

We observed 100% agreement in all assays tested, except HFE C282Y, which showed 99.75% agreement. The HFE C282Y assay was further confirmed through direct sequencing, and the results showed that OpenArray analysis was accurate. The calculated frequencies of each SNP were FV G1691A 98.8% (G/G), 1.2% (G/A); FII G2021A 99.5% (G/G), 0.5% (G/A); MTHFR C677T 45.5% (C/C), 44.8% (C/T), 9.8% (T/T); MTHFR A1298C 60.3% (A/A), 33.6% (A/C), 6.1% (C/C); HFE C282Y 96%(G/G), 4%(G/A), HFE H63D 78.1%(C/C), 20.3% (C/G), 1.6% (G/G); and HFE S65C 98.1% (A/A), 1.9% (A/T).

**Conclusion:**

Taken together, these results describe the frequencies of SNPs associated with diseases and are important to enhance our current knowledge of the genetic profiles of Brazilian blood donors, although a larger study is needed for a more accurate determination of the frequency of the alleles. Furthermore, the OpenArray platform showed a high concordance rate with standard FRET RT-PCR.

## Introduction

Blood donors are typically screened for the presence of infectious diseases to prevent transmission through blood transfusion. However, blood banks have recently begun testing for other factors, such as cholesterol and glucose levels, to evaluate the health of the donor and provide an incentive for blood donation [1, 2]. Blood typing has also become a routine test administered in blood banks [3, 4]. The presence of single nucleotide-polymorphisms (SNPs) associated with common genetic diseases could represent another factor for testing in blood donors, particularly if the cost of this test does not substantially increase the costs of tests routinely performed.

Genotyping tests for high throughput routines have being widely used in clinical laboratories. The OpenArray platform (Life Technologies, Carlsbad, CA) provides high density and requires a very low volume of sample based on nanoliters. It is a flexible system that uses TaqMan technology, capable of accommodating 3072 reactions per array [5].

Among the genetic factors related to diseases studied, Factor V Leiden (FV) (rs 6025) [6, 7] is the leading cause of genetic thrombophilia [8] and is observed in 5% of the Caucasian population [9]. The relative risk for venous thrombosis is 3–10 times higher for heterozygotes and 50–100 times higher for homozygotes carriers when compared to wild type subjects [8, 10]. The second most frequent genetic prothrombotic factor in humans is a mutation in prothrombin or coagulation factor II (rs1799963) [6, 7, 11]. Its prevalence in the Caucasian population is approximately 1 to 4%, and the frequency of this mutation among patients with venous thrombosis is 5 to 7% [11]. In addition, previous studies have indicated that the recurrence of venous thrombosis is higher for heterozygous individuals with mutations in FV Leiden and FII G20210A [12].

An increased level of homocysteine in plasma (hyperhomocysteinemia) also leads to prothrombotic events and is related to the presence of C677T (rs1801133) and A1298C (rs1801131) mutations [13–15]. Previous studies have reported an association between hemorrhagic (677TT and 677TT/1298AA genotypes) and ischemic stroke (1298CC and 677TT/1298CC genotypes) in cases of homozygous mutations, alone or in combination [16].

Another frequent disease also associated with SNPs is hereditary hemochromatosis (HH), an inherited disorder of iron metabolism resulting from mutations in the HFE gene. Clinically, HH is characterized as a multisystemic disease resulting in dysregulated intestinal iron absorption and progressive iron deposition in the liver, heart, skin, endocrine glands, and joints [17]. Thus, patients with HH might benefit from frequent blood donation [18, 19]. The most common genetic variants associated with HH are C282Y (rs1800562), H63D (rs1799945) and S65C (rs1800730) [20, 21]

In Brazil, the incidence of FV Leiden, FII G20210A and MTHFR C677T described in general population ranges from 0,7–4,8% for FV Leiden, 0,7–3,6% for FII 20210A and 35%- 44% for MTHFR C677T [22–29]. Few studies have evaluated the frequency of the HFE C282Y, H63D and S65C mutation in Brazilian healthy individuals The allelic frequencies described for HFE C282Y, H63D, S65C were 2,1%-2,3%, 13,6% and 0,6% respectively [30–32].

The aim of this study was to evaluate the OpenArray platform for genetic testing of blood donors. We also described the prevalence of these mutations among a Brazilian blood donor population.

## Materials and Methods

### Ethics Statements

A total of 400 blood samples were collected from October 24 to November 8, 2011, from blood donors at Fundação Pró-Sangue Hemocentro de São Paulo. There were 229 (52.3%) males and 171 (47.7%) females. The mean age of participants was 34.98 (SD±10.15) years-old. Participants were not classified into ethnic groups or skin color, as previous reports have shown genetic similarities among Brazilian population [33, 34]. Written informed consent was obtained from all participants. The study was approved by the Ethical Committee of the University São Paulo Medical School (asset n° 275/11), the ethical review board of Fundação Pró-Sangue Hemocentro de São Paulo.

### Controls

DNA samples with different alleles for all SNPs examined in this study were previously determined using a Real Time PCR—FRET system reaction and used as controls to validate the OpenArray method. The numbers of controls used for each SNP are described in Table 1.

**Table 1:** Comparison of the results obtained using OpenArray analysis and Light Cycler (LC).

Gene	SNP	Number of Controls for Genotyping	Number of controls with discordant Genotyping	Number of samples with Genotyping through OpenArray	Number of samples with discordant Genotyping through LC
Factor V Leiden	G1691A	19	0	392	0
Factor II	G20210A	18	0	390	0
MTHFR	C677T	24	0	388	0
MTHFR	A1298C	22	0	392	0
HFE	C282Y	22	1	392	1
HFE	H63 D	32	0	385	0
HFE	S65C	16	0	388	0

### DNA isolation

DNA was isolated from 200 μl of whole blood using the QIAamp DNA Mini kit (Qiagen, Hilden Germany), according to manufacturer’s instructions. The quantity and quality of the DNA was determined through spectrophotometry using a NanoDrop 2000 (Thermo Scientific, Wilmington, DE). All samples were diluted to a final concentration of 35 ng/μl.

### Genotyping

Genotyping was performed using TaqMan assays (Life Technologies). The rs numbers, mutation, gene and assay numbers for selected SNPs are described in Table 2.

**Table 2:** SNPs identification and Assay Part Numbers for OpenArray testing.

Gene	SNP	Rs number	Life Technologies Assay Part Number
Factor V Leiden	G1691A	rs6025	C__11975250_10
Factor II	G20210A	rs1799963	C___8726802_20
MTHFR	C677T	rs1801133	C___1202883_20
MTHFR	A1298C	rs1801131	C____850486_20
HFE	C282Y	rs1800562	C___1085595_10
HFE	H63 D	rs1799945	C___1085600_10
HFE	S65C	rs1800730	C___1085599_20

The assays included primers and probes labeled with VIC and FAM fluorophores, and each probe was designed for one particular allele type. The primers and probes were disposed using a solid platform (chip) that allows the performance of multiple samples and targets in the same reaction. The DNA and master mix were incorporated into the array using the OpenArray AccuFill System (Life Technologies), and the primers and probes were subsequently solubilized, followed by genotyping according to the manufacturer’s instructions. A non-template control (NTC), comprising DNase-free water, was introduced in each assay. The multiplex TaqMan assay reactions were performed using the GeneAmp PCR System 9700 (Life Technologies) with the following PCR cycle: an initial step at 93°C for 10 minutes, followed by 50 cycles of 45 seconds at 95°C, 13 seconds at 94°C and 2 minutes and 14 seconds at 53°C, with a final step at 25°C for 2 minutes and holding at 4°C.

The end point fluorescence was read using the BioTrove OpenArray SNP genotyping platform (Life Technologies). The genotyping analysis was performed using TaqMan Genotyper software version 1.3 (Life Technologies—available at http://www.lifetechnologies.com/br/en/home/global/forms/taqman-genotyper-softwaredownload-reg.html) with auto calling. The results were considered valid when the quality value call rate was 90% or higher.

The samples were classified as homozygous or heterozygous for alleles 1 or 2. The reproducibility of the OpenArray platform method was evaluated using 9 triplicate samples in three different runs. The accuracy was determined through a comparison of the genotyped results using Real-Time PCR—FRET methodology.

The real-time PCR-FRET (Fluorescence resonance energy transfer) was performed using a Light Cycler (LC) 2.0 (Roche Diagnostics, Meylan, France) instrument. The primers and probes for FV Leiden, FII and for HFE SNP detection have been previously described [35, 36]. The following commercial reagents were used for MTHFR C677T and A1298C: Light Mix C677T (Cat. No. 40-0095-16, TibMol Biol, Berlin, Germany) and Light Mix A1298C (Cat. No. 40-0269-16, TibMol Biol). The results of both methodologies were compared.

The inconsistent results were confirmed after sequencing the specific PCR product using the ABI Prism BigDye terminator kit v3.1 (Life Technologies) and the previously described primers [35–37] according to the manufacturer’s instructions. The reactions were run on an ABI Prism 3730 Genetic Analyzer (Life Technologies) and the results were analyzed using Sequencher—DNA Sequencing Analysis Software 4.1.4 (Life Technologies).

## Results

Among the blood donor samples tested, eight samples were excluded for call rates below 90%, as determined using Taqman Genotyper v1.3 software. Seven samples failed in at least one SNP, but because the call rates were higher than 90%, these samples were included in the analysis. None of the controls were excluded from the analysis.

Table 1 summarizes the comparison between the OpenArray system and the LC instrument. All blood donor and control samples had 100% agreement for all SNPs tested, except for HFE C282Y in two samples, which presented inconsistent results. One sample was classified as wild type using the OpenArray system and heterozygous using the LC, which showed an abnormal pattern of melting curve (the Tm observed was 51°C and 55°, while the standard Tm for heterozygous C282Y is 55°C and 64°C). This sample was further assessed using Sanger Sequencing, revealing a heterozygous silent mutation in another position (G843A instead of G845A). This finding was consistent with the result of the OpenArray system, as sequencing revealed that the sample was wild type for C282Y. The other sample was classified as heterozygous (G/A) using the OpenArray system and homozygous mutant (A/A) using the LC. Sequencing confirmed the OpenArray result.

To evaluate the reproducibility of the assay, nine samples were tested in triplicate, and no discrepancies were detected.

The genotypic and allelic frequencies of each SNP tested in the blood donor population were calculated using TaqMan Genotyper Software, and the results are shown in Table 3. The most frequent mutations found among the studied SNPs were C677T and A1298C in the MTHFR gene. Heterozygous genotypes were present in 44.8% (174/388) and 33.6% (132/392) of the blood donor samples tested for C677T and A1298C, respectively, while homozygous mutant genotypes were present in 9.8% (38/388) and 6.1% (24/392) of the samples, respectively. However, less frequent mutations were observed at Factor V (G1691A) and Factor II (G20210A), and these mutations were only present in heterozygous forms (1.2%- 5/392 for G1691A and 0.5%- 2/390 for G20210A).

**Table 3:** Genotypic and allelic frequencies observed in the studied population using the OpenArray method.

Gene/SNP	WT genotype	HET genotype	MUT genotype	Allele 1	Allele 2
FV G1691A	98,8% (G/G)	1,2% (G/A)	0% (A/A)	99,4% (G)	0,6% (A)
FII G20210A	99,5% (G/G)	0,5%(G/A)	0% (A/A)	0,2% (A)	99,8%(G)
MTHF C677T	45,5% (C/C)	44,8% (C/T)	9,8% (T/T)	67,9% (C)	32,1% (T)
MTHF A1298C	60,3% (A/A)	33,6% (A/C)	6,1% (C/C)	22,9% (C)	77,1% (A)
HFE C282Y	96,0%(G/G)	4,0%(G/A)	0% (A/A)	98,0% (G)	2,0% (A)
HFE H63D	78,1%(C/C)	20,3%(C/G)	1,6(G/G)	86,9% (C)	13,1 (G)
HFE S65C	98,1% (A/A)	1,9% (A/T)	0%(T/T)	99,0% (A)	1,0%(T)

WT—Wild Type; HET—Heterozygous; MUT- Homozygous for the mutation.

## Discussion

The OpenArray genotyping method was used to simultaneously detect seven different mutations in genes associated with thrombophilia and hemochromatosis. For evaluation purposes, the results were compared with FRET based real time PCR and a high concordance level was observed between methods, with only a single discrepancy in 2,727 determinations. Further studies may confirm the accurace of OpenArray method.

In the present study, we found that the frequencies of the heterozygous genotype for FV Leiden (G1691A) and FII (G20210A) were low, and the homozygous FV 1691AA and FII 20210AA genotype were completely absent among the studied group. Similar results were reported previously in Brazilian healthy subjects [22–24]. These data suggest that the prevalence of FV Leiden and FII G20210A, associated with thrombophilic events, is lower in Brazilian blood donors than that reported in previous studies concerning blood donors of other countries [38–40]. For HFE mutations (C282Y, H63D and S65C), associated with an iron overload in homozygous or compound genotypes characteristic of HH, we observed a higher frequency (20.3% heterozygous and 1.6% homozygous) of H63D, consistent with previous studies reported internationally [21] and in a Brazilian population of blood donors from São Paulo [41]. We did not observe the presence of a homozygous genotype for either C282Y or S65C among the studied population, and the heterozygous compound genotype C282Y/H63D was observed in only one sample. The MTHFR mutations showed a prevalence of 44.8% (C/T) and 9.8% (T/T) for C677T and 33.6% (A/C) and 6.1% (C/C) for A1298C, consistent with the results obtained from previous studies in Brazilian children and other control groups [22–24, 26, 42]. A comparison of the results obtained in the present study with those obtained in a recent prevalence study in middle-southern Italian blood donors revealed that the cohort used in this study presented a higher number of wild-type subjects for both C677T and A1298C [43]. We also observed that none of the individuals tested carried a 677TT/1298CC genotype. Because some SNPs have not been found in mutant homozygous state and show a very low frequency in heterozygous state, such as FV G1691A (1.2%), FII G20210A (0.5%), HFE C282Y (4%) and HFE S65C (1.9%), the screening of a larger number of samples is required for a more accurate determination of the frequency of the alleles in blood donors from São Paulo, Brazil.

Penetrance is defined as the proportion of individuals of a particular genotype who express the corresponding phenotype [44]. Although we found that some subjects carry the mutant allele, they may not express the disorder due the reduced (or incomplete) penetrance showed by some autosomal dominant and recessive inherited mutations, such as FV G1691A and HFE C282Y respectively. Genotyping studies of apparently healthy individuals may be an approach to understand the penetrance of pathological variants [45].

Genetic testing may also be used for the identification of the FV Leiden mutation among women. Hormone therapies that primarily contain estrogens enhance the risk of thrombosis among this population [46, 47]. Although, current guidelines consider genetic testing to identify carriers of high-risk thrombophilia are only worthwhile when the subject has a family history of venous thromboembolism [48], the identification of this deficiency could provide more information to weigh the risks and benefits of hormonal contraceptive therapy in young women and hormone replacement in menopausal women. Indeed, further studies may contribute to certainly define whether offering genetic tests to blood donors may be beneficial.

In conclusion, the OpenArray methodology showed good concordance of results when compared with FRET based real time PCR. Although a larger study is require to have a more accurate frequency of the alleles, the frequency of SNPs related with thrombophilias and hemochromatosis in Brazilian blood donors are very similar with results previously reported by other groups.

## References

[1] Goette L, Stutzer A, Yavuzcan G, Frey BM. Free cholesterol testing as a motivation device in blood donations: evidence from field experiments. Transfusion. 2009;49(3):524–31. 10.1111/j.1537-2995.2008.02007.x . 19040493

[2] Lenhard MJ, Maser RE, Kolm P, Healy MJ, Seshadri P. Screening blood donors for diabetes: analysis of use, accuracy, and cost. Transfusion. 2013;53(11):2776–81. 10.1111/trf.12135 . 23451798

[3] Westhoff CM. Molecular testing for transfusion medicine. Current opinion in hematology. 2006;13(6):471–5. 10.1097/01.moh.0000245695.77758.3d . 17053461

[4] Anstee DJ. Red cell genotyping and the future of pretransfusion testing. Blood. 2009;114(2):248–56. 10.1182/blood-2008-11-146860 . 19411635

[5] Morrison T, Hurley J, Garcia J, Yoder K, Katz A, Roberts D, et al Nanoliter high throughput quantitative PCR. Nucleic acids research. 2006;34(18):e123 10.1093/nar/gkl639 17000636 PMC1635282

[6] Bezemer ID, Bare LA, Doggen CJ, Arellano AR, Tong C, Rowland CM, et al Gene variants associated with deep vein thrombosis. JAMA: the journal of the American Medical Association. 2008;299(11):1306–14. 10.1001/jama.299.11.1306 . 18349091

[7] Delluc A, Gourhant L, Lacut K, Mercier B, Audrezet MP, Nowak E, et al Association of common genetic variations and idiopathic venous thromboembolism. Results from EDITh, a hospital-based case-control study. Thrombosis and haemostasis. 2010;103(6):1161–9. 10.1160/TH09-07-0430 . 20352152

[8] Bauduer F, Lacombe D. Factor V Leiden, prothrombin 20210A, methylenetetrahydrofolate reductase 677T, and population genetics. Molecular genetics and metabolism. 2005;86(1–2):91–9. 10.1016/j.ymgme.2005.04.002 . 16185908

[9] Endler G, Mannhalter C. Polymorphisms in coagulation factor genes and their impact on arterial and venous thrombosis. Clin Chim Acta. 2003;330(1–2):31–55. Epub 2003/03/15. S0009898103000226 [pii]. . 12636925 10.1016/s0009-8981(03)00022-6

[10] Bertina RM, Koeleman BP, Koster T, Rosendaal FR, Dirven RJ, de Ronde H, et al Mutation in blood coagulation factor V associated with resistance to activated protein C. Nature. 1994;369(6475):64–7. 10.1038/369064a0 . 8164741

[11] Poort SR, Rosendaal FR, Reitsma PH, Bertina RM. A common genetic variation in the 3'-untranslated region of the prothrombin gene is associated with elevated plasma prothrombin levels and an increase in venous thrombosis. Blood. 1996;88(10):3698–703. . 8916933

[12] De Stefano V, Martinelli I, Mannucci PM, Paciaroni K, Chiusolo P, Casorelli I, et al The risk of recurrent deep venous thrombosis among heterozygous carriers of both factor V Leiden and the G20210A prothrombin mutation. The New England journal of medicine. 1999;341(11):801–6. 10.1056/NEJM199909093411104 . 10477778

[13] Eldibany MM, Caprini JA. Hyperhomocysteinemia and thrombosis: an overview. Archives of pathology & laboratory medicine. 2007;131(6):872–84. 10.1043/1543-2165(2007)131[872:HATAO]2.0.CO;2 . 17550314

[14] Vares M, Saetre P, Deng H, Cai G, Liu X, Hansen T, et al Association between methylenetetrahydrofolate reductase (MTHFR) C677T polymorphism and age of onset in schizophrenia. American journal of medical genetics Part B, Neuropsychiatric genetics: the official publication of the International Society of Psychiatric Genetics. 2010;153B(2):610–8. 10.1002/ajmg.b.31030 . 19746410

[15] Jacques PF, Bostom AG, Williams RR, Ellison RC, Eckfeldt JH, Rosenberg IH, et al Relation between folate status, a common mutation in methylenetetrahydrofolate reductase, and plasma homocysteine concentrations. Circulation. 1996;93(1):7–9. . 8616944 10.1161/01.cir.93.1.7

[16] Sazci A, Ergul E, Tuncer N, Akpinar G, Kara I. Methylenetetrahydrofolate reductase gene polymorphisms are associated with ischemic and hemorrhagic stroke: Dual effect of MTHFR polymorphisms C677T and A1298C. Brain research bulletin. 2006;71(1–3):45–50. 10.1016/j.brainresbull.2006.07.014 . 17113927

[17] Alexander J, Kowdley KV. HFE-associated hereditary hemochromatosis. Genetics in medicine: official journal of the American College of Medical Genetics. 2009;11(5):307–13. 10.1097/GIM.0b013e31819d30f2 . 19444013

[18] Leitman SF. Hemochromatosis: the new blood donor. Hematology / the Education Program of the American Society of Hematology American Society of Hematology Education Program. 2013;2013:645–50. 10.1182/asheducation-2013.1.645 . 24319245

[19] Pennings G. Demanding pure motives for donation: the moral acceptability of blood donations by haemochromatosis patients. Journal of medical ethics. 2005;31(2):69–72. 10.1136/jme.2002.001271 15681668 PMC1734081

[20] Davis CF, Dorak MT. An extensive analysis of the hereditary hemochromatosis gene HFE and neighboring histone genes: associations with childhood leukemia. Annals of hematology. 2010;89(4):375–84. 10.1007/s00277-009-0839-y . 19806355

[21] Spinola C, Brehm A, Spinola H. Prevalence of H63D, S65C, and C282Y hereditary hemochromatosis gene variants in Madeira Island (Portugal). Annals of hematology. 2011;90(1):29–32. 10.1007/s00277-010-1034-x . 20714725

[22] Dalmaz CA, Santos KG, Botton MR, Tedoldi CL, Roisenberg I. Relationship between polymorphisms in thrombophilic genes and preeclampsia in a Brazilian population. Blood cells, molecules & diseases. 2006;37(2):107–10. 10.1016/j.bcmd.2006.07.005 . 16963292

[23] de Paula Sabino A, Guimaraes DA, Ribeiro DD, Paiva SG, Sant'Ana Dusse LM, das Gracas Carvalho M, et al Increased Factor V Leiden frequency is associated with venous thrombotic events among young Brazilian patients. Journal of thrombosis and thrombolysis. 2007;24(3):261–6. 10.1007/s11239-007-0024-x . 17401546

[24] Dusse LM, Carvalho M, Braganca WF, Paiva SG, Godoi LC, Guimaraes DA, et al Inherited thrombophilias and pre-eclampsia in Brazilian women. European journal of obstetrics, gynecology, and reproductive biology. 2007;134(1):20–3. 10.1016/j.ejogrb.2006.09.006 . 17097210

[25] Lima MB, de Oliveira-Filho AB, Campos JF, Melo FC, Neves WB, Melo RA, et al Increased risk of venous thrombosis by AB alleles of the ABO blood group and Factor V Leiden in a Brazilian population. Genetics and molecular biology. 2009;32(2):264–7. 10.1590/S1415-47572009000200010 21637678 PMC3036915

[26] Stur E, Silveira AN, Selvatici LS, Alves LN, de Vargas Wolfgramm E, Tovar TT, et al Polymorphism analysis of MTHFR, factor II, and factor V genes in the Pomeranian population of Espirito Santo, Brazil. Genetic testing and molecular biomarkers. 2012;16(3):219–22. 10.1089/gtmb.2011.0163 . 21919702

[27] Filho IL, Leite AC, Moura PG, Ribeiro GS, Cavalcante AC, Azevedo FC, et al Genetic polymorphisms and cerebrovascular disease in children with sickle cell anemia from Rio de Janeiro, Brazil. Arquivos de neuro-psiquiatria. 2011;69(3):431–5. . 21755116 10.1590/s0004-282x2011000400004

[28] Ramos CPS, Campos JF, Neves WB, Santos ME, Araujo FA, Melo RAM. Frequency of factor V Leiden in individuals under thrombophilia investigation, Recife, Pernambuco, Brazil. Rev bras Hematol Hemoter. 2006;28(2):131–4.

[29] Ramos CPS, Campos JF, Neves WB, Santos ME, Araujo FA, Melo RAM. Mutant prothrombin in individuals under thrombophilia investigation. J Bras Patol Med Lab. 2008;44(2):79–82.

[30] Santos PC, Cancado RD, Terada CT, Rostelato S, Gonzales I, Hirata RD, et al HFE gene mutations and iron status of Brazilian blood donors. Brazilian journal of medical and biological research = Revista brasileira de pesquisas medicas e biologicas / Sociedade Brasileira de Biofisica [et al]. 2010;43(1):107–14. . 20027482 10.1590/s0100-879x2009007500031

[31] Santos PC, Krieger JE, Pereira AC. Molecular diagnostic and pathogenesis of hereditary hemochromatosis. International journal of molecular sciences. 2012;13(2):1497–511. 10.3390/ijms13021497 22408404 PMC3291973

[32] Terada CT, Santos PC, Cancado RD, Rostelato S, Lopreato FR, Chiattone CS, et al Iron deficiency and frequency of HFE C282Y gene mutation in Brazilian blood donors. Transfusion medicine. 2009;19(5):245–51. 10.1111/j.1365-3148.2009.00944.x . 19747287

[33] Pena SD, Di Pietro G, Fuchshuber-Moraes M, Genro JP, Hutz MH, Kehdy Fde S, et al The genomic ancestry of individuals from different geographical regions of Brazil is more uniform than expected. PloS one. 2011;6(2):e17063 10.1371/journal.pone.0017063 21359226 PMC3040205

[34] Santos RV, Fry PH, Monteiro S, Maio MC, Rodrigues JC, Bastos-Rodrigues L, et al Color, race, and genomic ancestry in Brazil: dialogues between anthropology and genetics. Current anthropology. 2009;50(6):787–819. . 20614657 10.1086/644532

[35] van den Bergh FA, van Oeveren-Dybicz AM, Bon MA. Rapid single-tube genotyping of the factor V Leiden and prothrombin mutations by real-time PCR using dual-color detection. Clinical chemistry. 2000;46(8 Pt 1):1191–5. . 10926904

[36] Bernard PS, Ajioka RS, Kushner JP, Wittwer CT. Homogeneous multiplex genotyping of hemochromatosis mutations with fluorescent hybridization probes. The American journal of pathology. 1998;153(4):1055–61. 10.1016/S0002-9440(10)65650-7 9777937 PMC1853057

[37] Madjunkova S, Volk M, Peterlin B, Plaseska-Karanfilska D. Detection of thrombophilic mutations related to spontaneous abortions by a multiplex SNaPshot method. Genetic testing and molecular biomarkers. 2012;16(4):259–64. 10.1089/gtmb.2011.0173 22023244 PMC3326265

[38] Kvasnicka J, Hajkova J, Bobcikova P, Kvasnicka T, Duskova D, Poletinova S, et al Prevalence of thrombophilic mutations of FV Leiden, prothrombin G20210A and PAl-1 4G/5G and their combinations in a group of 1450 healthy middle-aged individuals in the Prague and Central Bohemian regions (results of FRET real-time PCR assay). Cas Lek Cesk. 2012;151(2):76–82. . 22515013

[39] Lee DH, Henderson PA, Blajchman MA. Prevalence of factor V Leiden in a Canadian blood donor population. CMAJ. 1996;155(3):285–9. 8705907 PMC1487985

[40] Sottilotta G, Mammi C, Furlo G, Oriana V, Latella C, Trapani Lombardo V. High incidence of factor V Leiden and prothrombin G20210A in healthy southern Italians. Clin Appl Thromb Hemost. 2009;15(3):356–9. 10.1177/1076029607310218 . 19211580

[41] Santos PC, Pereira AC, Cancado RD, Schettert IT, Sobreira TJ, Oliveira PS, et al HFE gene mutations in patients with primary iron overload: is there a significant improvement in molecular diagnosis yield with HFE sequencing? Blood cells, molecules & diseases. 2010;45(4):302–7. 10.1016/j.bcmd.2010.08.008 . 20843714

[42] Alessio AC, Annichino-Bizzacchi JM, Bydlowski SP, Eberlin MN, Vellasco AP, Hoehr NF. Polymorphisms in the methylenetetrahydrofolate reductase and methionine synthase reductase genes and homocysteine levels in Brazilian children. American journal of medical genetics Part A. 2004;128A(3):256–60. 10.1002/ajmg.a.30108 . 15216546

[43] Zappacosta B, Graziano M, Persichilli S, Di Castelnuovo A, Mastroiacovo P, Iacoviello L. 5,10-Methylenetetrahydrofolate reductase (MTHFR) C677T and A1298C polymorphisms: genotype frequency and association with homocysteine and folate levels in middle-southern Italian adults. Cell biochemistry and function. 2014;32(1):1–4. 10.1002/cbf.3019 . 24277487

[44] Beutler E, Felitti VJ, Koziol JA, Ho NJ, Gelbart T. Penetrance of 845G—> A (C282Y) HFE hereditary haemochromatosis mutation in the USA. Lancet. 2002;359(9302):211–8. 10.1016/S0140-6736(02)07447-0 . 11812557

[45] Cooper DN, Krawczak M, Polychronakos C, Tyler-Smith C, Kehrer-Sawatzki H. Where genotype is not predictive of phenotype: towards an understanding of the molecular basis of reduced penetrance in human inherited disease. Human genetics. 2013;132(10):1077–130. 10.1007/s00439-013-1331-2 23820649 PMC3778950

[46] Mira Y, Aznar J, Estelles A, Vaya A, Villa P, Ferrando F. Congenital and acquired thrombotic risk factors in women using oral contraceptives: clinical aspects. Clin Appl Thromb Hemost. 2000;6(3):162–8. . 10898277 10.1177/107602960000600308

[47] Aznar J, Cerda G. Factor V Leiden carriers taking oral contraceptives have an increased risk of thrombosis. American journal of obstetrics and gynecology. 2013;209(2):156 10.1016/j.ajog.2013.02.036 . 23453804

[48] De Stefano V, Rossi E. Testing for inherited thrombophilia and consequences for antithrombotic prophylaxis in patients with venous thromboembolism and their relatives. A review of the Guidelines from Scientific Societies and Working Groups. Thrombosis and haemostasis. 2013;110(4):697–705. 10.1160/TH13-01-0011 . 23846575

